# Supplementary material for: Recent SARS-CoV-2 infection abrogates antibody and B-cell responses to booster vaccination
Source: medRxiv. 2022 Aug 31:2022.08.30.22279344. Preprint. [Version 1] doi: 10.1101/2022.08.30.22279344 (PMC9460969; doi:10.1101/2022.08.30.22279344)
Supplement: Supplement 1 [file media-1.pdf]

SUPPLEMENTAL FIGURES AND TABLES

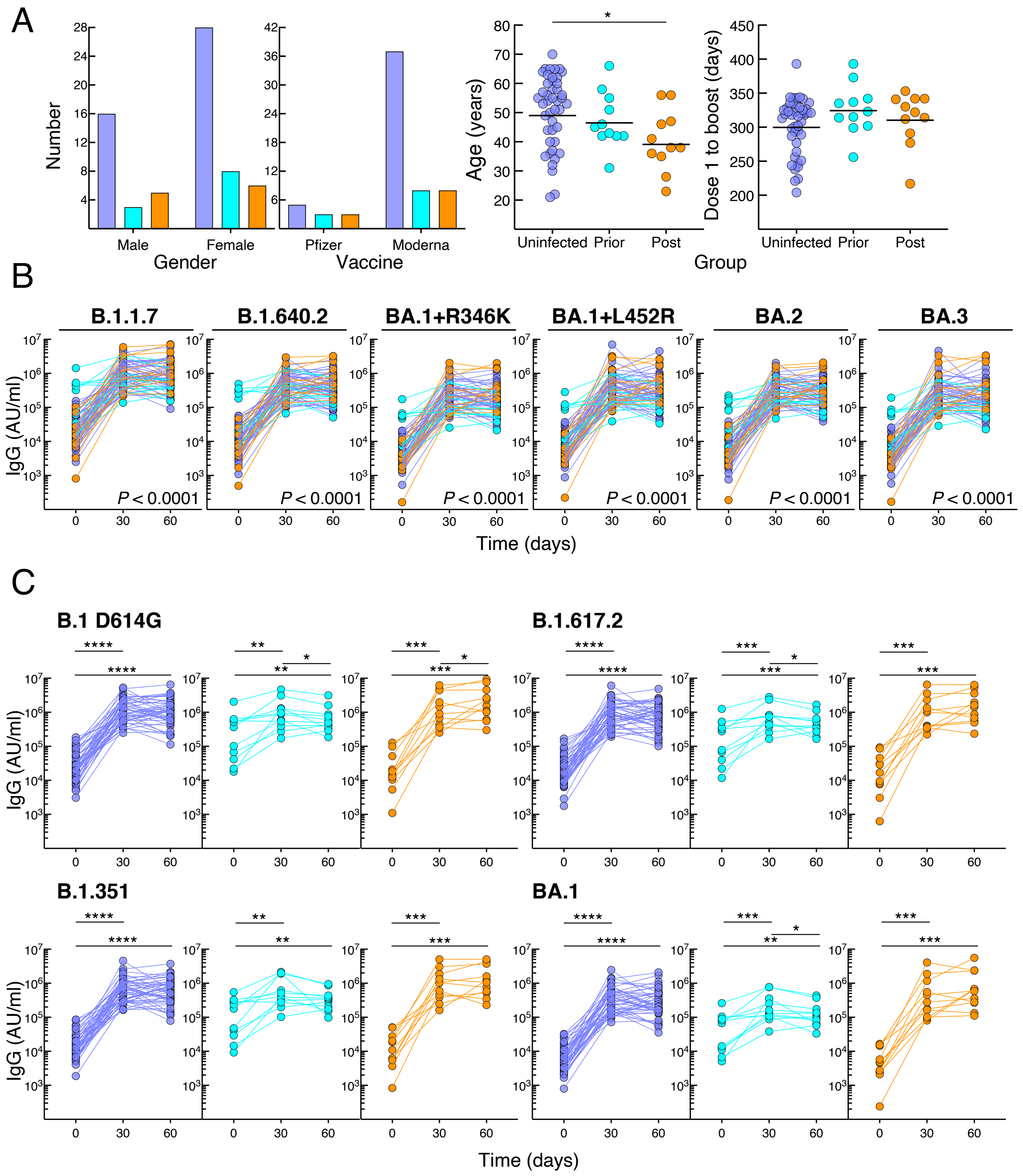

D

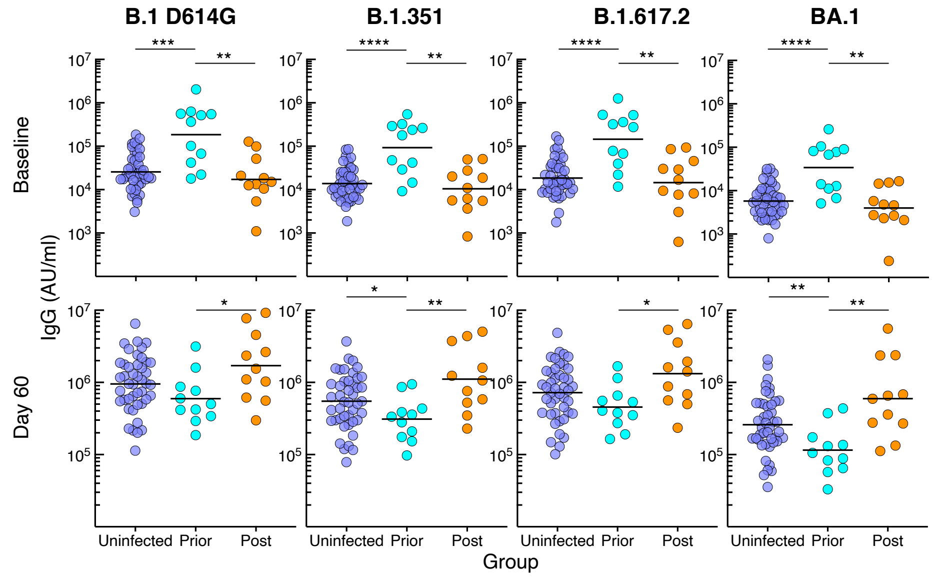

B.1 D614G
B.1.351
B.1.617.2
BA.1
Baseline
Day 60
IgG (AU/ml)
Uninfected Prior Post
Group

E

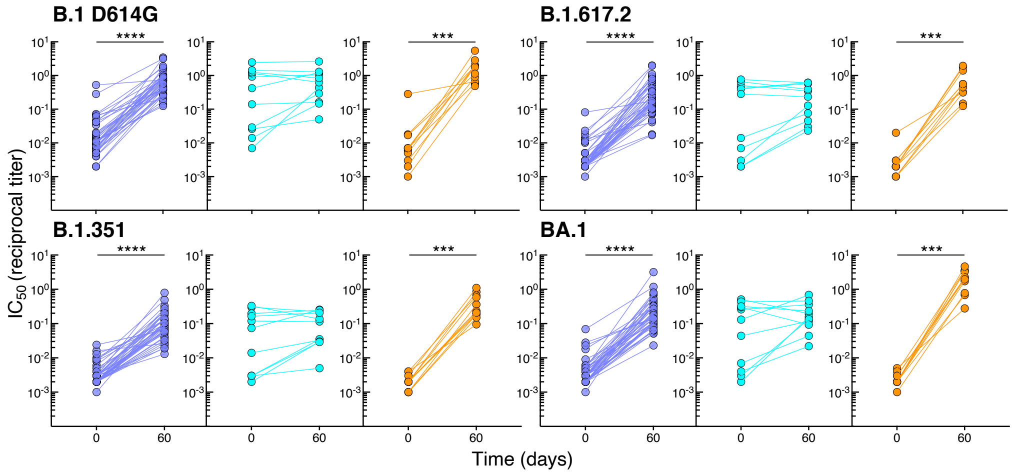

B.1 D614G
B.1.617.2
B.1.351
BA.1
IC_{50} (reciprocal titer)
Time (days)

F

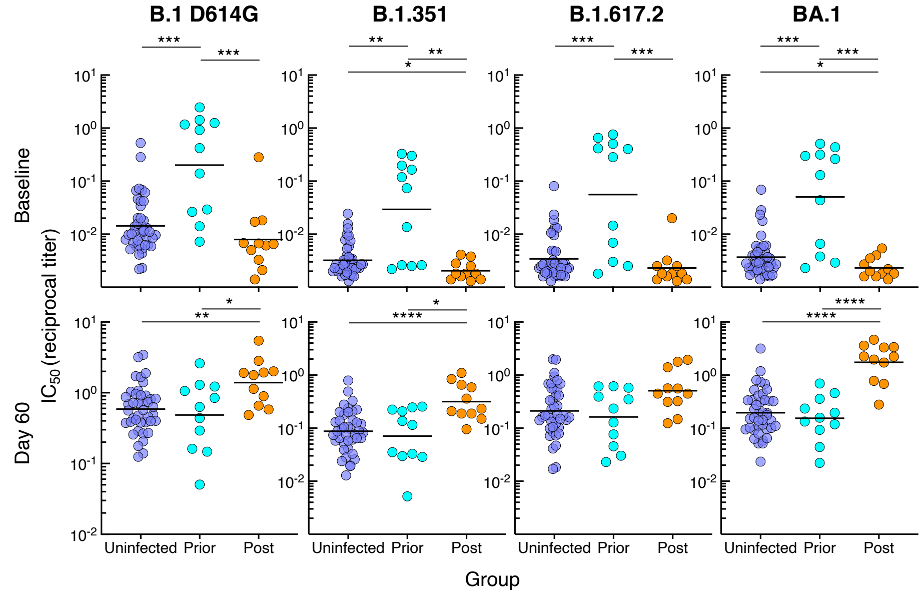

B.1 D614G
B.1.351
B.1.617.2
BA.1
Baseline
Day 60
IC_{50} (reciprocal titer)
Uninfected Prior Post
Group

Figure S1. Demographics and antibody responses to booster vaccination

(A) Comparison between the groups for gender, vaccine, age, and interval between dose 2 and booster vaccination.

(B) Serum IgG spike-binding titers for additional variants, expressed as in Figure 1B.

(C) Serum IgG spike-binding titers in Figure 1B shown by group.

(D) Comparison between the groups at baseline and day 60 of serum IgG spike-binding titers in Figure 1B.

(E) Serum neutralizing titers in Figure 1D shown by group.

(F) Comparison between the groups at baseline and day 60 of serum neutralizing titers in Figure 1D.

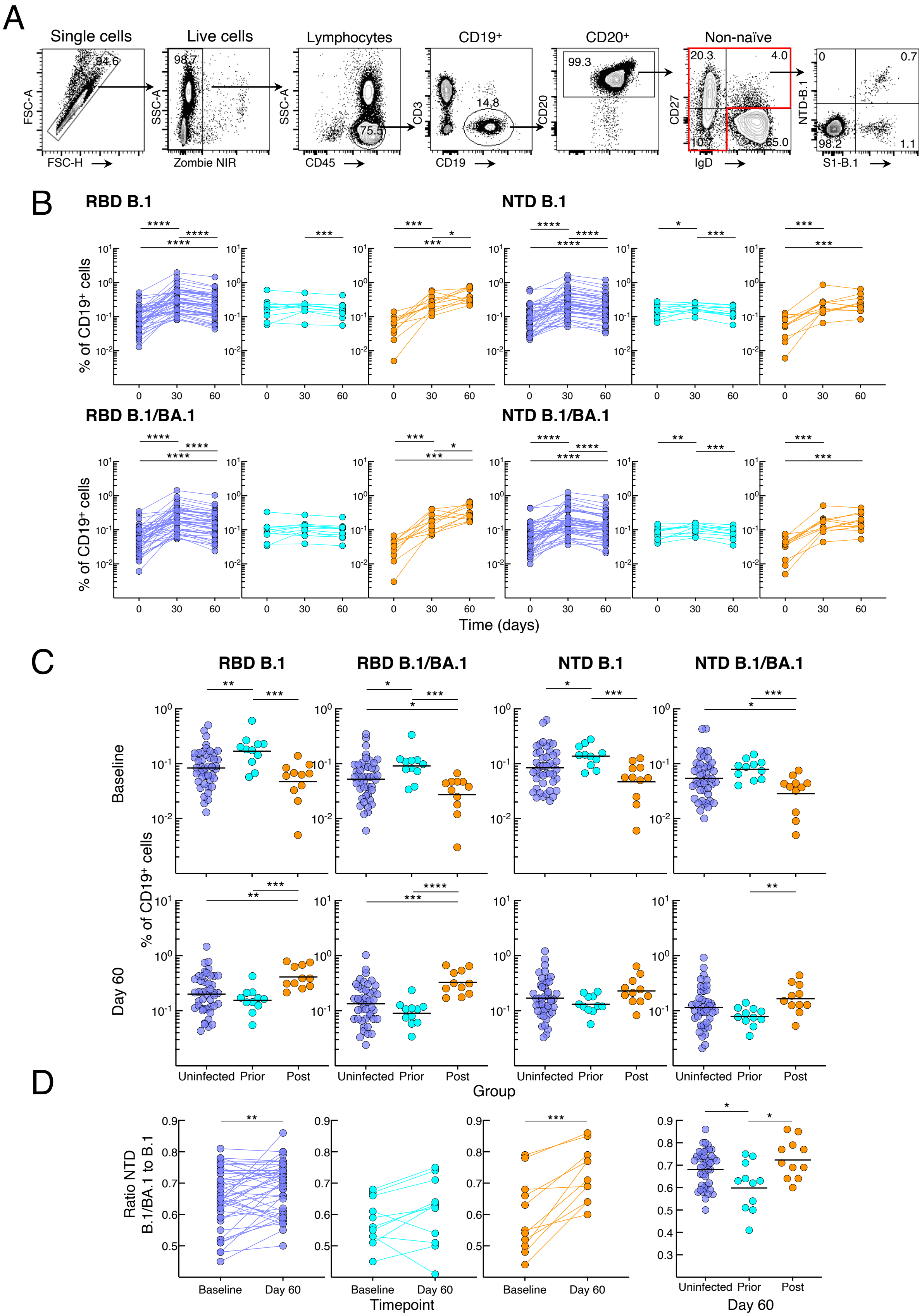
A
Single cells
Live cells
Lymphocytes
CD19+
CD20+
Non-naïve
FSC-A
FSC-H
SSC-A
Zombie NIR
CD45
CD3
CD19
CD20
CD27
IgD
NTD-B.1
S1-B.1
B
RBD B.1
NTD B.1
RBD B.1/BA.1
NTD B.1/BA.1
% of CD19+ cells
Time (days)
C
Baseline
Day 60
Uninfected
Prior
Post
Group
D
Ratio NTD B.1/BA.1 to B.1
Baseline
Day 60
Timepoint

Figure S2. Spike-binding B cells

(A) Gating strategy for identifying non-naïve B cells; also showing representative binding of B.1 NTD and S1 tetramers.

(B) Frequencies of B.1 and BA.1 RBD and NTD tetramer-binding B cells in Figure 2B shown by group.

(C) Comparison between the groups at baseline and day 60 of frequencies of B.1 and BA.1 RBD and NTD tetramer-binding B cells in Figure 2B.

(D) As in Figure 2D but for NTD.

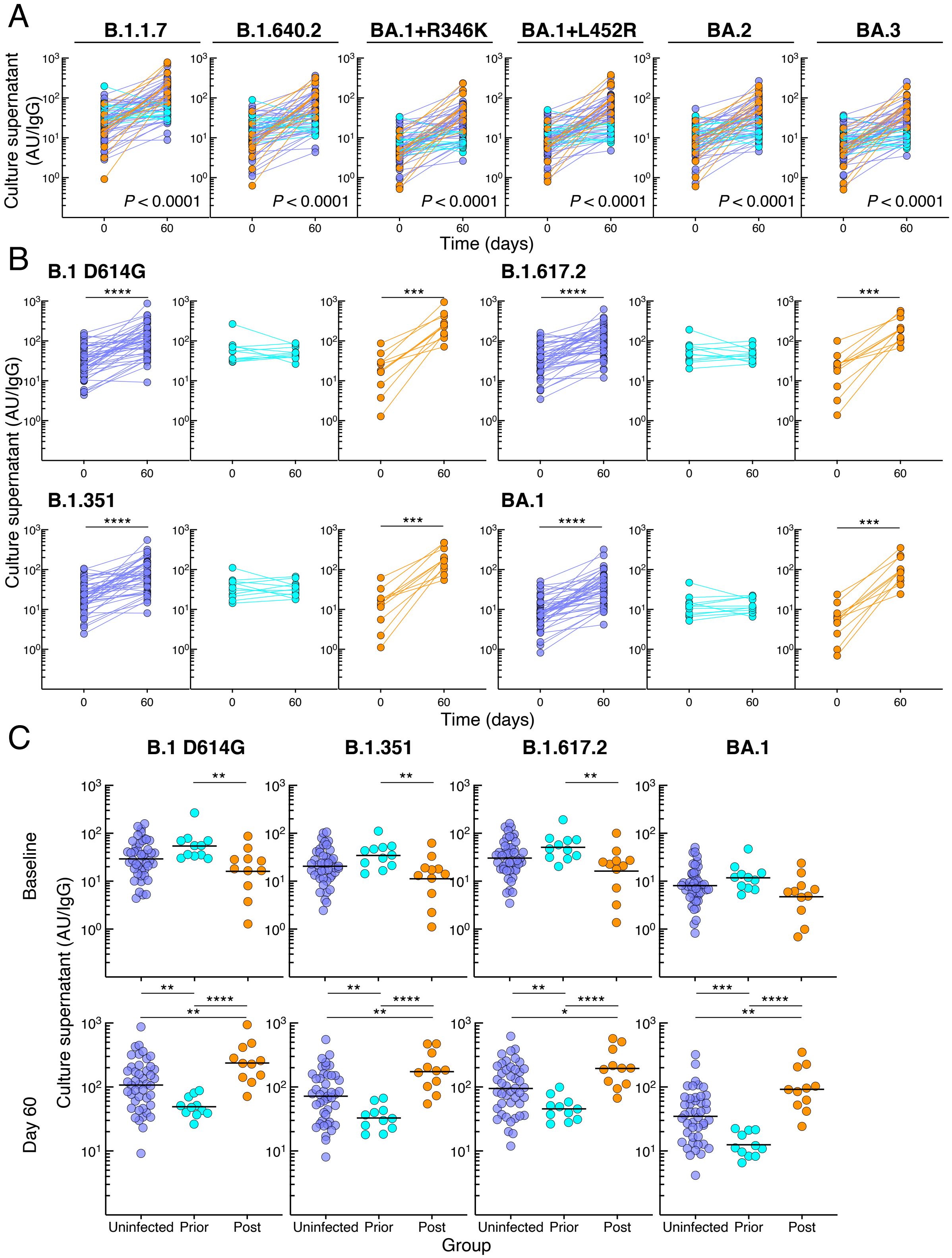

A
B.1.1.7
B.1.640.2
BA.1+R346K
BA.1+L452R
BA.2
BA.3
Culture supernatant (AU/IgG)
P < 0.0001
Time (days)
B
B.1 D614G
B.1.617.2
B.1.351
BA.1
Culture supernatant (AU/IgG)
Time (days)
C
B.1 D614G
B.1.351
B.1.617.2
BA.1
Baseline
Day 60
Culture supernatant (AU/IgG)
Uninfected
Prior
Post
Group

Figure S3. Spike-binding antibodies secreted from B cells in cultured PBMCs

(A) Secreted IgG spike-binding titers for additional variants, expressed as in Figure 3A.

(B) Secreted IgG spike-binding titers in Figure 3A shown by group.

(C) Comparison between the groups at baseline and day 60 of secreted IgG spike-binding titers in Figure 3A.

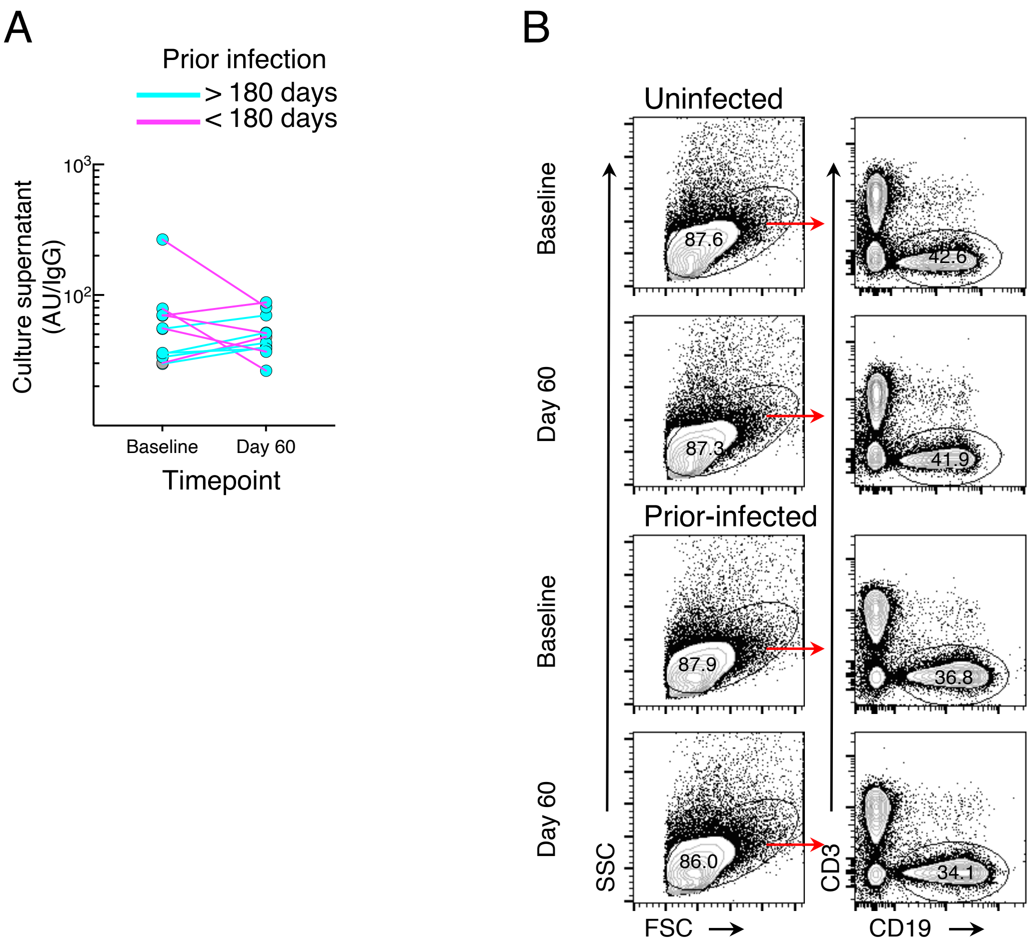

Figure S4. *In vitro* proliferation studies

(A) Secreted antibodies in Figure S3B for the prior-infected group against B.1 D614G spike shown color-coordinated by time of infection relative to booster vaccination. PBMCs of the six individuals with interval < 180 days were stimulated to evaluate cell division by CFSE dilution.

(B) Gating of live $CD19^+$ cells after four days in culture.

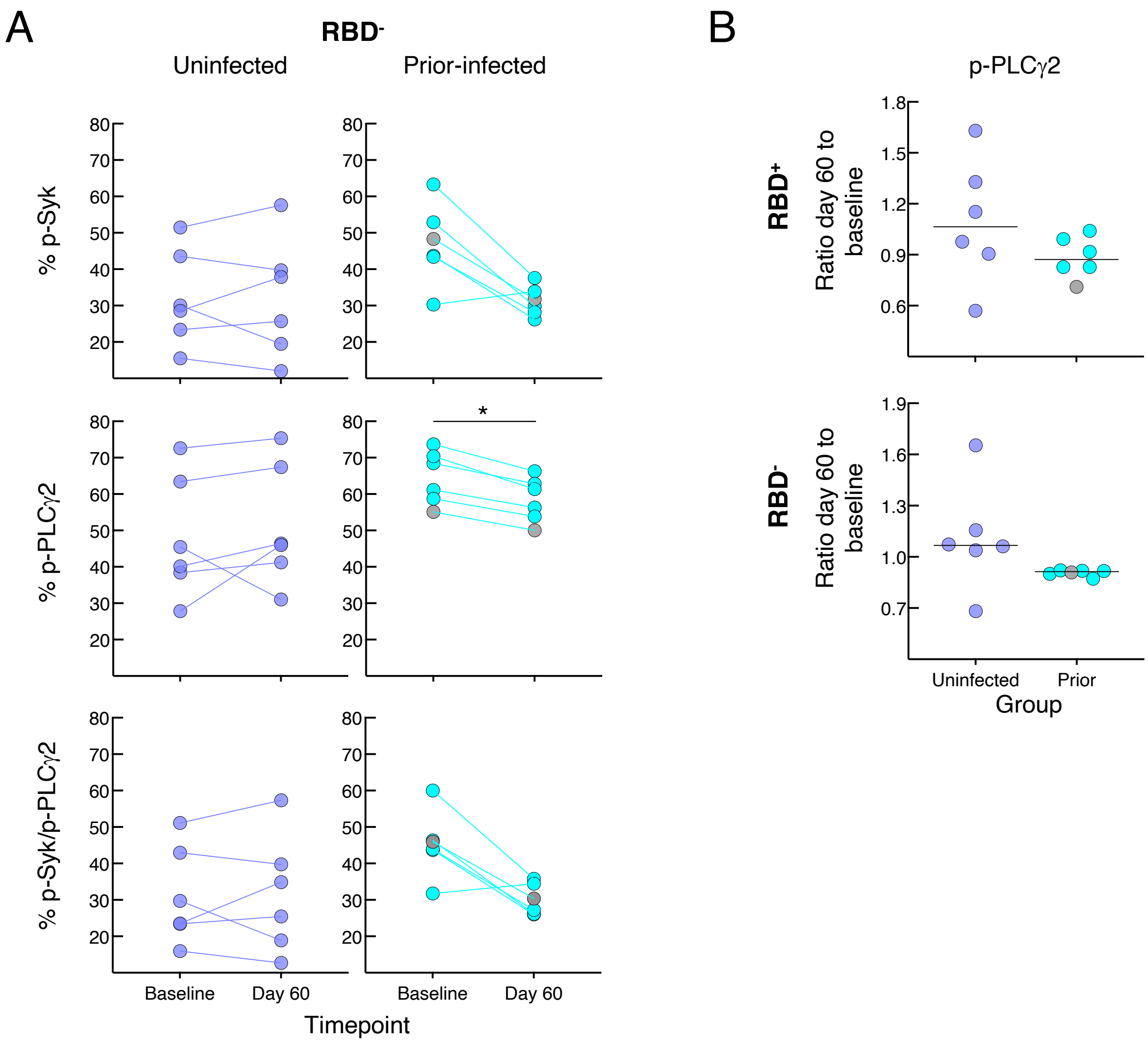

Figure S5. ***In vitro*** **BCR signaling studies**

(A) As in Figure 5B but for RBD⁻ B cells.

(B) As in Figure 5C but for single PLCγ2.

Table S1: *P* values for serum binding antibody increase over time or between group pairs

Variant	Unin[a] day 0-30	Unin day 0-60	Unin day 30-60	Prior infected day 0-30	Prior infected day 0-60	[b]Prior infected day 30-60	Post infected day 0-30	Post infected day 0-60	Post infected day 30-60	Prior vs unin day 0	[c]Prior vs unin day 60	Prior vs Post day 0	[c]Prior vs Post day 60	Post vs unin day 0	Post vs unin day 60	Day 60:0 fold unin vs prior	Day 60:0 fold post vs unin	Day 60:0 fold post vs prior
B.1.1.7	<0.0001	<0.0001	ns	0.002	0.002	ns	0.001	0.001	0.0186	<0.0001	ns	0.0014	0.0128	ns	ns	<0.0001	0.0080	<0.0001
B.1.640.2	<0.0001	<0.0001	ns	0.0029	0.0186	0.042	0.001	0.001	0.0322	0.0001	ns	0.0014	0.0052	ns	0.0457	<0.0001	0.0060	<0.0001
BA.1 R346K	<0.0001	<0.0001	ns	0.001	0.0049	0.0098	0.001	0.001	ns	0.0001	0.0065	0.0032	0.0024	ns	ns	<0.0001	0.0174	<0.0001
BA.1 L452R	<0.0001	<0.0001	ns	0.001	0.0029	ns	0.001	0.001	ns	<0.0001	0.0164	0.0032	0.0024	ns	ns	<0.0001	0.0222	<0.0001
BA.2	<0.0001	<0.0001	ns	0.002	0.0049	0.0322	0.001	0.001	0.0244	<0.0001	ns	0.0014	0.0041	ns	0.0249	<0.0001	0.0145	<0.0001
BA.3	<0.0001	<0.0001	ns	0.001	0.0049	0.0137	0.001	0.001	ns	<0.0001	0.008	0.0024	0.0024	ns		<0.0001	0.0174	<0.0001

[a]Uninfected

[b]Decreased from previous timepoint

[c]Decreased vs uninfected or post-infected

Table S2: *P* values for group by time interaction for mixed-effects model of spike-binding serum antibody titers

Variant	All	Prior- vs post-infected	Uninfected vs prior-infected	Uninfected vs post-infected
B.1 D614G	<0.0001	<0.0001	<0.0001	0.1589
B.1.1.7	<0.0001	<0.0001	<0.0001	0.0706
B.1.315	<0.0001	<0.0001	<0.0001	0.1589
B.1.617.2	<0.0001	<0.0001	<0.0001	0.1589
B.1.640.2	<0.0001	<0.0001	<0.0001	0.0683
BA.1	<0.0001	<0.0001	<0.0001	0.0683
BA.1 R346K	<0.0001	<0.0001	<0.0001	0.1034
BA.1 L452R	<0.0001	<0.0001	<0.0001	0.1589
BA.2	<0.0001	<0.0001	<0.0001	0.1034
BA.3	<0.0001	<0.0001	<0.0001	0.1589

Table S3: *P* Values for group by time interaction for mixed-effects model of frequencies of spike-binding B cells

Spike tetramer	All	Prior- vs post-infected	Uninfected vs prior-infected	Uninfected vs post-infected
B.1 S1	<0.0001	<0.0001	<0.0001	<0.0001
B.1 RBD	<0.0001	<0.0001	<0.0001	<0.0001
B.1 NTD	<0.0001	<0.0001	<0.0001	0.0002
B.1.& BA.1 RBD	<0.0001	<0.0001	<0.0001	<0.0001
B.1.& BA.1 NTD	<0.0001	<0.0001	<0.0001	0.0001

Table S4: *P* values for secreted antibody increase over time or between group pairs

Variant	Unin[a] day 0-36	Prior infected day 0-60	Post infected day 0-60	Prior vs unin day 0	[b]Prior vs unin day 60	Prior vs post day 0	[b]Prior vs post day 60	Post vs unin day 0	Post vs unin day 60	Day 60:0 fold unin vs prior	Day 60:0 fold post vs unin	Day 60:0 fold post vs prior
B.1.1.7	<0.0001	ns	0.001	0.0154	0.007	0.0024	<0.0001	ns	0.0029	<0.0001	0.0002	<0.0001
B.1.640.2	<0.0001	ns	0.001	0.0220	0.006	0.0052	<0.0001	ns	0.0045	<0.0001	0.0004	<0.0001
BA.1 R346K	<0.0001	ns	0.001	ns	0.0001	ns	<0.0001	ns	0.0060	<0.0001	0.0001	<0.0001
BA.1 L452R	<0.0001	ns	0.001	ns	0.0006	ns	<0.0001	ns	0.0039	<0.0001	0.0002	<0.0001
BA.2	<0.0001	ns	0.001	ns	0.0031	ns	<0.0001	ns	0.0031	<0.0001	0.0007	<0.0001
BA.3	<0.0001	ns	0.001	ns	0.0006	ns	<0.0001	ns	0.0033	<0.0001	0.0001	<0.0001

[a]Uninfected

[b]Decreased vs uninfected or post-infected

Table S5: *P* values for group by time interaction for mixed-effects model of spike-binding secreted antibody titers

Variant	All	Prior- vs post-infected	Uninfected vs prior-infected	Uninfected vs post-infected
B.1 D614G	<0.0001	<0.0001	<0.0001	0.0001
B.1.1.7	<0.0001	<0.0001	<0.0001	<0.0001
B.1.315	<0.0001	<0.0001	<0.0001	0.0001
B.1.617.2	<0.0001	<0.0001	<0.0001	0.0001
B.1.640.2	<0.0001	<0.0001	<0.0001	0.0001
BA.1	<0.0001	<0.0001	<0.0001	<0.0001
BA.1 R346K	<0.0001	<0.0001	<0.0001	0.0001
BA.1 L452R	<0.0001	<0.0001	<0.0001	0.0001
BA.2	<0.0001	<0.0001	<0.0001	0.0001
BA.3	<0.0001	<0.0001	<0.0001	0.0001

Table S6: 21-color flow cytometry panel

Reagent	Source
Mouse anti-human CD45 BUV805 (clone HI30)	BD Biosciences
Mouse anti-human CD19 BV650 (Clone SJ25-C1)	BD Biosciences
Mouse anti-human CD20 APC-H7 (Clone 2H7)	BD Biosciences
Mouse anti-human CD10 BV510 (Clone HI10a)	BD Biosciences
Mouse anti-human IgG PE-Cy7 (Clone G18-145)	BD Biosciences
Mouse anti-human CD11c BUV395 (clone B-Ly6)	BD Biosciences
Mouse anti-human CD3 BV570 (Clone UCHT1)	Biolegend
Mouse anti-human IgD BV605 (Clone IA6-2)	Biolegend
Mouse anti-human IgM BV711 (Clone MHM-88)	Biolegend
Mouse anti-human CD27 BV785 (Clone O323)	Biolegend
Mouse anti-human CD21 PE/Dazzle594 (Clone BU32)	Biolegend
Mouse anti-human CD38 APC/Fire810 (Clone HB-7)	Biolegend
Mouse anti-human CD71 Alexa Fluor 700 (Clone CY1G4)	Biolegend
Mouse anti-human IgA VioBlue (Clone IS11-8E10)	Miltenyi Biotec
SARS-CoV-2 S1 B.1 (#793806)	Biolegend
SARS-CoV-2 Spike B.1 trimer (#SPN-C82E9)	AcroBiosystems
SARS-CoV-2 RBD B.1 (#SPD-C82E9)	AcroBiosystems
SARS-CoV-2 RBD BA.1 (#SPD-C82E4)	AcroBiosystems
SARS-CoV-2 S NTD B.1	In-house
SARS-CoV-2 S NTD BA.1	In-house
Streptavidin PE	ThemoFisher
Streptavidin APC	ThemoFisher
Streptavidin PE-Cy5.5	ThemoFisher
Streptavidin Alexa Fluor 488	ThemoFisher
Streptavidin BV421	Biolegend
Streptavidin BUV615	BD Biosciences
Zombie NIR Fixable Viability Dye	Biolegend
